# Moth bean (*Vigna aconitifolia*): a minor legume with major potential to address global agricultural challenges

**DOI:** 10.3389/fpls.2023.1179547

**Published:** 2023-06-06

**Authors:** Kanishka R. C., Gayacharan, Basavaraja T., Rahul Chandora, Jai Chand Rana

**Affiliations:** ^1^ Indian Council of Agricultural Research (ICAR)-National Bureau of Plant Genetic Resources, Regional Station, Shimla, India; ^2^ Indian Council of Agricultural Research (ICAR)-National Bureau of Plant Genetic Resources, New Delhi, India; ^3^ Indian Council of Agricultural Research (ICAR)-Indian Institute of Pulses Research, Kanpur, India; ^4^ Alliance of Bioversity International and International Center for Tropical Agriculture (CIAT), India Office, National Agricultural Science Complex, New Delhi, India

**Keywords:** moth bean, genetic diversity, crop improvement, stress tolerance, climate change, sustainable agriculture

## Abstract

Moth bean (*Vigna aconitifolia*) is an orphan legume of *Vigna* genus, exhibiting wide adaptability and has the potential to grow well in arid and semi-arid areas, predominantly across different eco-geographical regions of Asia, particularly the Indian subcontinent. The inherent adaptive attributes of this crop have made it more tolerant towards a diverse array of abiotic and biotic stresses that commonly restrain yield among other *Vigna* species. Additionally, the legume is recognized for its superior nutritional quality owing to its high protein content as well as amino acid, mineral and vitamin profile and is utilized as both food and fodder. Moth bean can play a vital role in sustaining food grain production, enhancing nutritional security as well as provide a source of income to resource-poor farmers amid rise in global temperatures and frequent drought occurrences, particularly in rain-fed cropping systems which accounts for about 80% of the world’s cultivated land. However, this minor legume has remained underutilized due to over-exploitation of major staple crops. With the exception of a few studies involving conventional breeding techniques, crop improvement in moth bean for traits such as late maturity, indeterminate growth habit, shattering and anti-nutritional factors has not garnered a lot of attention. Recent advances in sequencing technologies, modern breeding approaches and precision phenotyping tools, in combination with the available crop gene pool diversity in gene banks, can accelerate crop improvement in moth bean and lead to the development of improved cultivars. Considering the recent surge in awareness about the development of climate-smart crops for sustainable agricultural future, collective effort towards effective utilization of this hardy, neglected legume is the need of the hour.

## 1 Introduction

Agricultural sustainability is stymied by excessive reliance on major staple crops, climate change as well as deterioration of cultivable land. These limitations extend a challenge to global food security while simultaneously accentuating rural poverty and malnutrition in developing and under-developed nations. Many crops are missing from agricultural fields while others have seen a slump in both cultivation and usage. Evolution of new pests and pathogens along with enhanced crop uniformity in farmers’ field are posing a serious threat to crop production. Tackling global food demand and hidden hunger warrants a radical shift from current unsustainable agricultural practices and call attention to alternative potential future crops. Introduction of new crop species in cropping systems and enhancing crop diversity through the utilization of diverse germplasm can play vital role in global food and nutritional security. Underutilized plants are the potential genetic resources to be exploited to address the decreasing nutritional quality and stress resilience in the already mainstreamed cultivars. ‘Underutilized’ plant species are those that had been grown widely in the past or have the capability to be grown extensively in the future, however, are cultivated restrictively owing to agronomic, economic or genetic causes (Gruère et al., 2006). They are also known as ‘orphan’ or ‘neglected’ crops owing to insufficient information available about them as a result of sparse consideration from research and development (Eyzaguirre et al., 1999). These crops remain ‘underexploited’ or ‘minor’ since their economic potential has not been ascertained (Padulosi and Hoeschle-Zeledon, 2004).

Pulse crops are exceptional sources of quality protein, in addition to being utilized for their medicinal properties, as fodder for livestock, for enriching soil as a result of symbiotic relationship with nitrogen-fixing bacteria and mitigating greenhouse emissions (Lemke et al., 2007; Singh et al., 2007; Stagnari et al., 2017). The current scenario of booming food insecurity signals an urgency to reformulate and steer crop improvement and production agronomy strategies, in the coming decades, towards grain legumes to successfully determine climate-resilient species having enhanced grain attributes (Considine et al., 2017). Crop diversification, alternative cropping systems and value-added products’ development are significant contributions made by underutilized legumes, to the life of local communities. Mostly, these minor pulses are adapted to marginal lands. The genetic resources of these orphan plants face rapid destruction owing to changes in traditional food habits and depreciation of traditional farming culture along with the introduction and acclimatization of high yielding crops. Therefore, mainstreaming of these underutilized crop plants, essentially minor legumes, should be set in motion in order to impede global food concerns.

**Figure 1:**
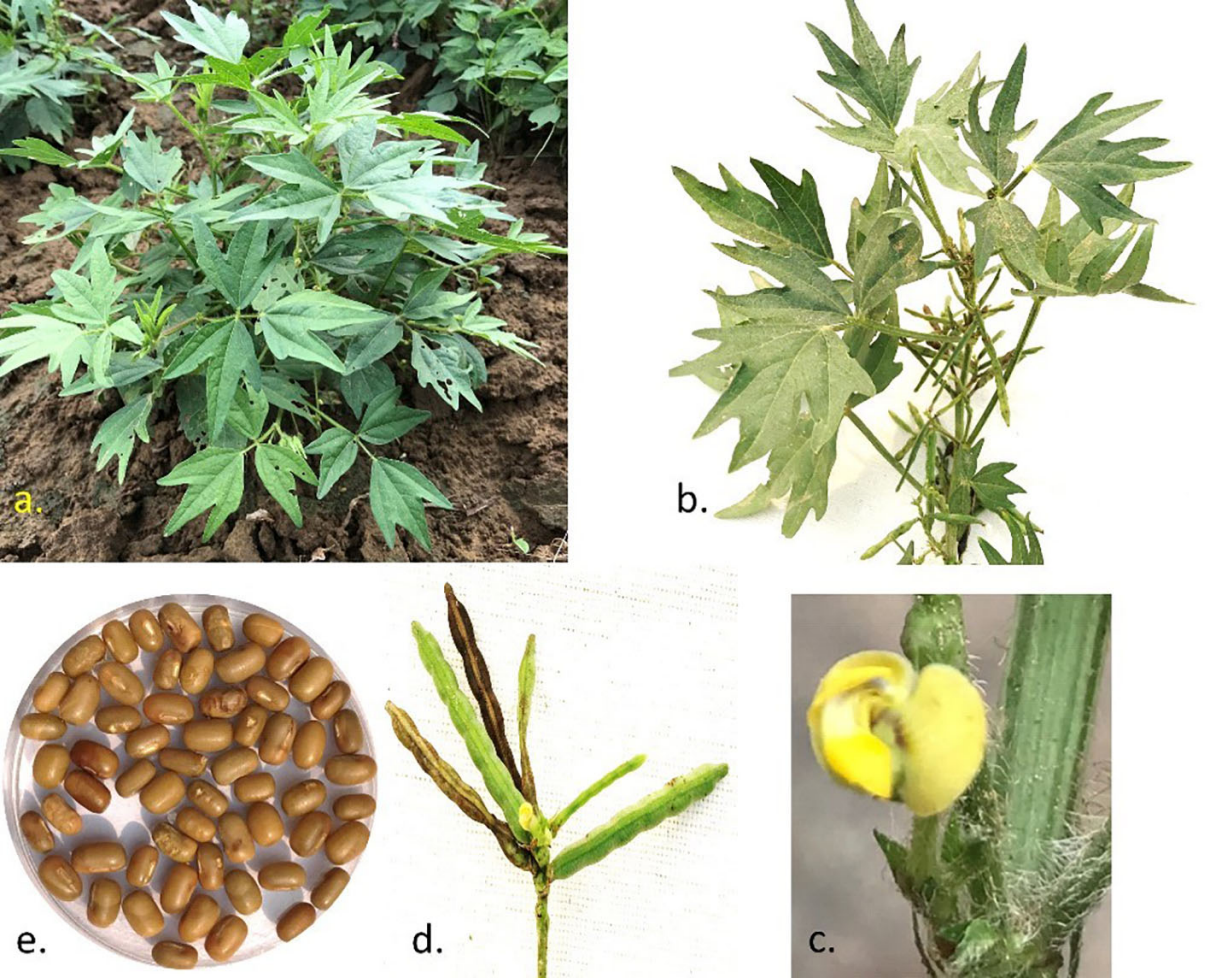
Highlights of the morphological features of Moth bean: A typical plant type with central branch and trailing primary branches **(A)**, Inflorescence and pods in a branch **(B)**, papilionaceous flower **(C)**, semi-erect pods arrangement in peduncle **(D)** and seed shape and size **(E)**. Seeds are placed in a 2.5 cm dish.

Moth bean [*Vigna aconitifolia* (Jacq.) Maréchal syn. *Phaseolus aconitifolius* Jacq. (2n= 2x=22)] is an underutilized, minor grain legume. It is also known as mat bean, math, mattenbohne, matki, dew bean, Turkish gram or haricot papillon (Heuzé et al., 2020). Moth bean is an annual herbaceous trailing legume of warm and dry habitats, particularly the semi-arid and arid regions of Indian subcontinent. It is principally grown for its protein-rich seeds, sprouts and green pods that are used as vegetable, apart from being equally important as feed and fodder for livestock and as a cover crop as well as green manuring, hence serves as multipurpose crop (Saravanan and Ignacimuthu, 2015) (**Figure 1**). Integrated consumption of moth bean like pulse legume crops with cereals is expedient as they have a complementary relationship, for moth is rich in lysine and leucine and cereals supply sulphur-containing amino acids, thereby supplementing mutual amino acid deficiencies.

**Figure 2:**
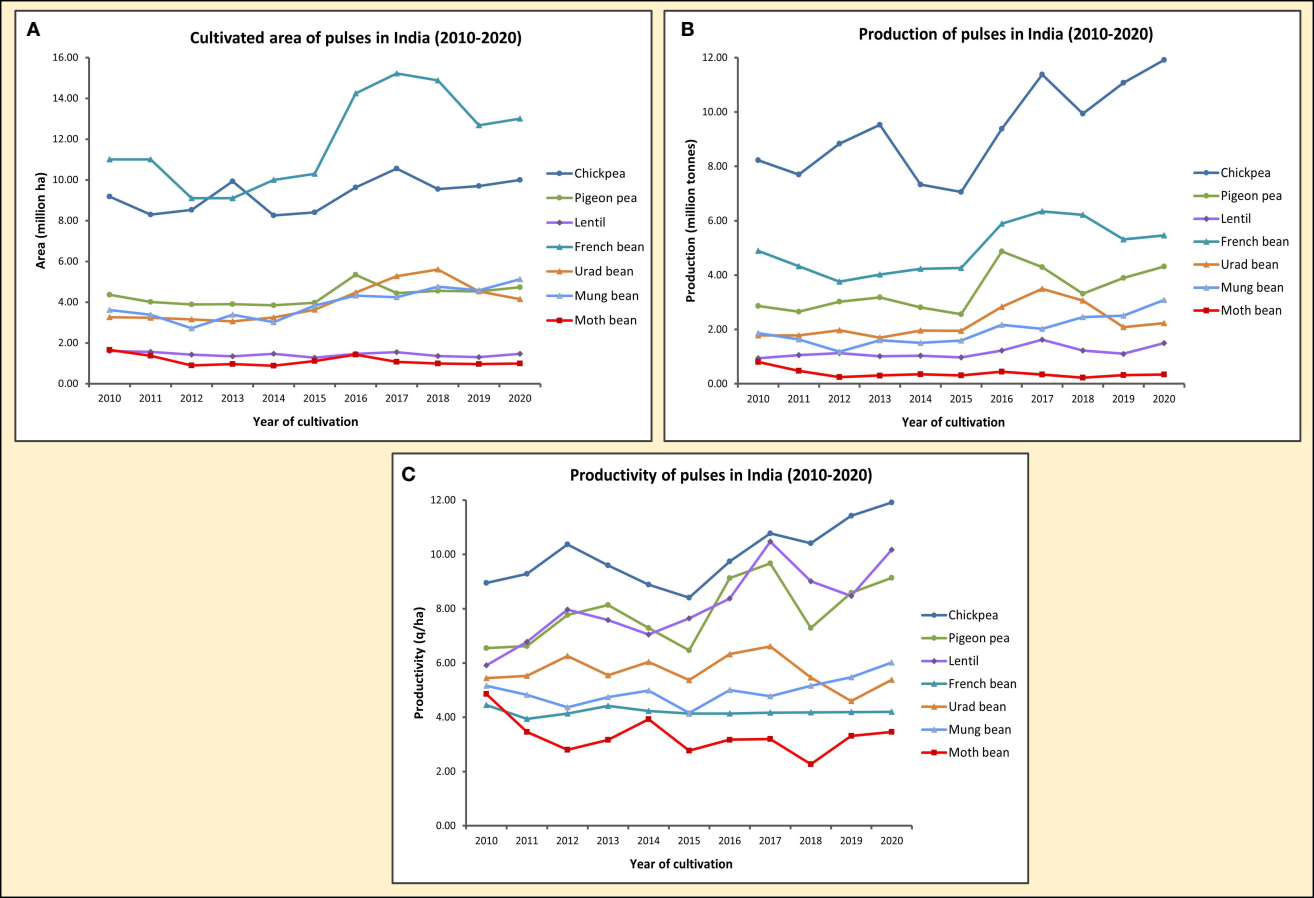
Graphs showing the cultivated area in million ha **(A)**, production in million tonnes **(B)** and productivity in q/ha **(C)** of seven pulse crops, including Moth bean, in India from 2010-2020. [Source: Directorate of Economics and Statistics, India (eands.dacnet.nic.in); Food and Agriculture Organization Corporate Statistical Database (FAOSTAT) (www.fao.org/faostat)].

As a prospective major legume crop, *Vigna aconitifolia* is quite notable for being the most drought hardy and heat tolerant pulse among Asian *Vigna*s (Tomooka et al., 2011). It is usually grown on marginal and sub-marginal lands with poor moisture holding capacity. Having a deep, fast penetrating tap root system and a dense low-lying leaf cover that resembles a mat, it can withstand lack of water and drying, hot temperatures (∼ 40°C), by keeping the soil moist as well as lowering soil temperature, thereafter also reducing the possibility of soil erosion. The multi-adaptive nature of moth bean, in addition to requiring little to no care and input, has led it to being recognized as an arid legume. Notwithstanding the attributes of moth bean that makes it a future legume for sustainable agriculture, it is only grown limitedly and there has been a precipitous reduction in the cultivable area and production of the legume over the past decade (**Figure 2**) owing to agronomic bottlenecks *viz.*, low variability, slow growth, longer maturation duration, substandard yields due to poor seed set and below par response to fertilizer treatments (Harsh et al., 2016). Its competence, with respect to agronomy, genetics and nutrition has been vastly overlooked. This review attempts to encapsulate the work done in *Vigna aconitifolia*, in an effort to harness its potentialities to curb the unprecedented challenges to global agriculture.

## 2 Origin and distribution

Moth bean is considered to be a native of India, Pakistan, Myanmar and Sri Lanka (Piper and Morse, 1914; Purseglove, 1974; Rachie and Roberts, 1974; Jain and Mehra, 1980). Vavilov (1926) and De Candolle (1986) reported India as the center of origin for wild and cultivated forms of *V. aconitifolia* (Jacq), and Maréchal (1978) proposed that Sri Lanka and Pakistan are the centers of diversity of this minor legume. Dana (1976) proffered that the wild forms of moth bean were distributed from Sonoran Desert of Mexico to Tapachula, near the Guatemalan border in Central America.

**Figure 3:**
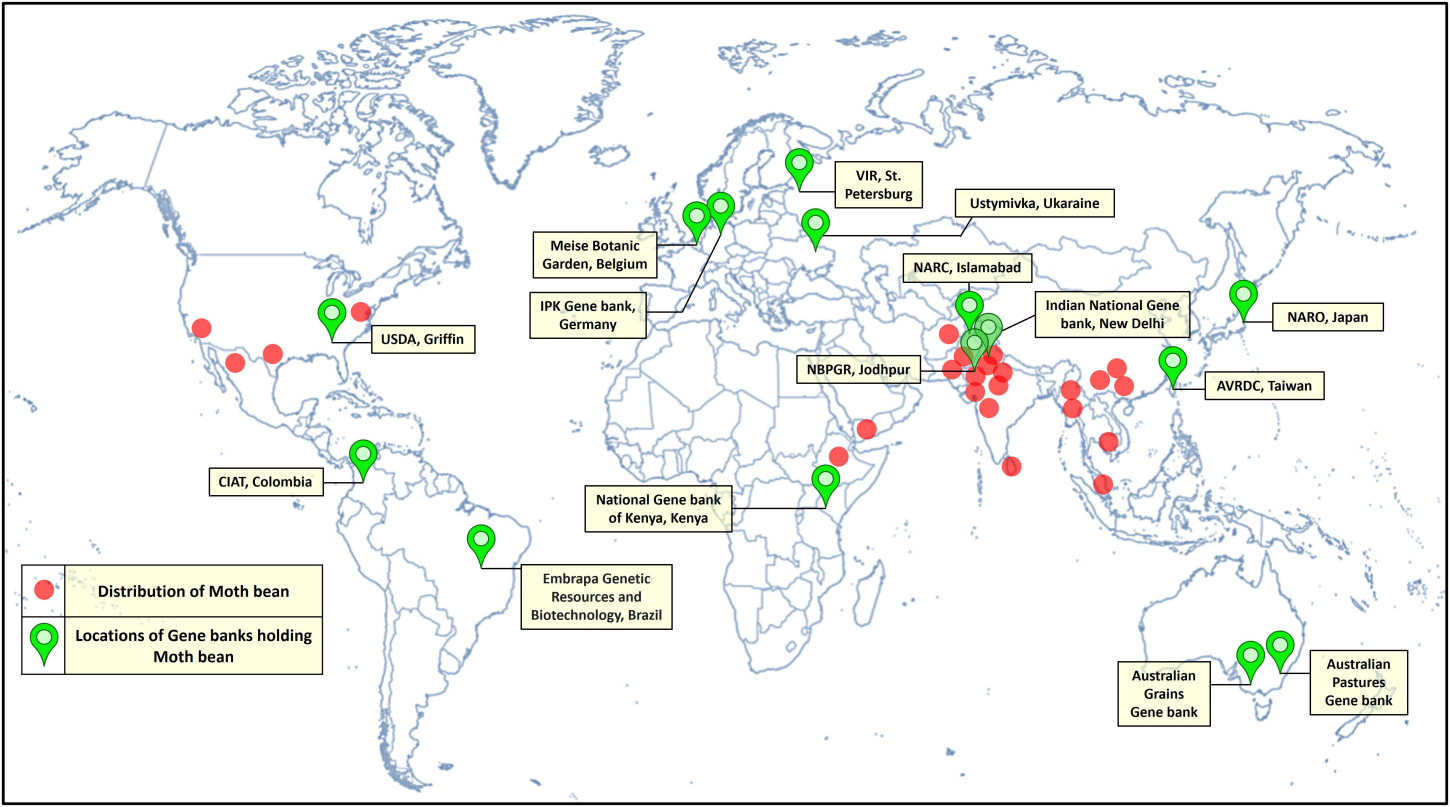
Distribution and locations of moth bean germplasm holdings across the world.

*Vigna aconitifolia* is principally cultivated in India. It is also grown in Sri Lanka, Myanmar, China, Pakistan, Malaysia, Africa and is also grown in the South-Western states of the USA (Jain and Mehra, 1980; Munro and Small, 1998; Brink and Jansen, 2006; Kochhar, 2016). In India, it is distributed from the North-West Himalayas (up to the height of 6000 m) to Karnataka in south and from the foot hills of North-East Himalayas in the east to Saurashtra in the west (Arora, 1985). Moth bean is essentially grown in the arid and semi-arid regions, particularly in the North-Western states. Rajasthan, Maharashtra, Gujarat, Punjab, Haryana, Jammu and Kashmir, Madhya Pradesh and Uttar Pradesh are the prime moth bean growing states (Arora, 1985) (**Figure 3**). Rajasthan, the driest state in India, contributes the most, both in terms of production as well as area, at national level (Gupta et al., 2016; Viswanatha et al., 2016).

## 3 Taxonomy and phylogenetic relationship

Moth bean belongs to the genus *Vigna* and sub-family Papilionoideae which comes under the family Fabaceae. It is considered as one of the most primitive species among the *Vigna* genus, with respect to its evolution (Smartt, 1985). Earlier, moth bean or Dew gram (*Phaseolus aconitifolius* Jacq. syn. *Vigna aconitifolia* (Jacq.) Marechal) was a part of the genus *Phaseolus*. Thereafter, it was shifted to the *Vigna* genus of the tribe Phaseolae. *Vigna* and *Phaseolus*, together, form a very elaborate taxonomic group called the *Phaseolus-Vigna* complex. Verdcourt (1970) proposed that the genus *Phaseolus* be restricted to exclusively include those American species that have a tightly coiled style and pollen grains without course reticulation, thereby significantly promoting the concept of *Vigna*. In the taxonomic revision made by Verdcourt (1970) in Phaseolus Linn. and Vigna savi, species with yellow-colored flowers, under subgenus *Ceratotropis* Piper, were transferred to *Vigna* genus, *Ceratotropis* Verdc. Subgenus. This led to an increase in the number of species included in the genus *Vigna*. Maréchal (1978) followed Verdcourt by presenting a monograph on the *Phaseolus-Vigna* complex and also proposed nomenclatural changes, following which, *Phaseolus aconitifolius* (Jacq.) became *Vigna aconitifolia* (Jacq.) Marechal. This taxonomic system is generally accepted now.

**Figure 4:**
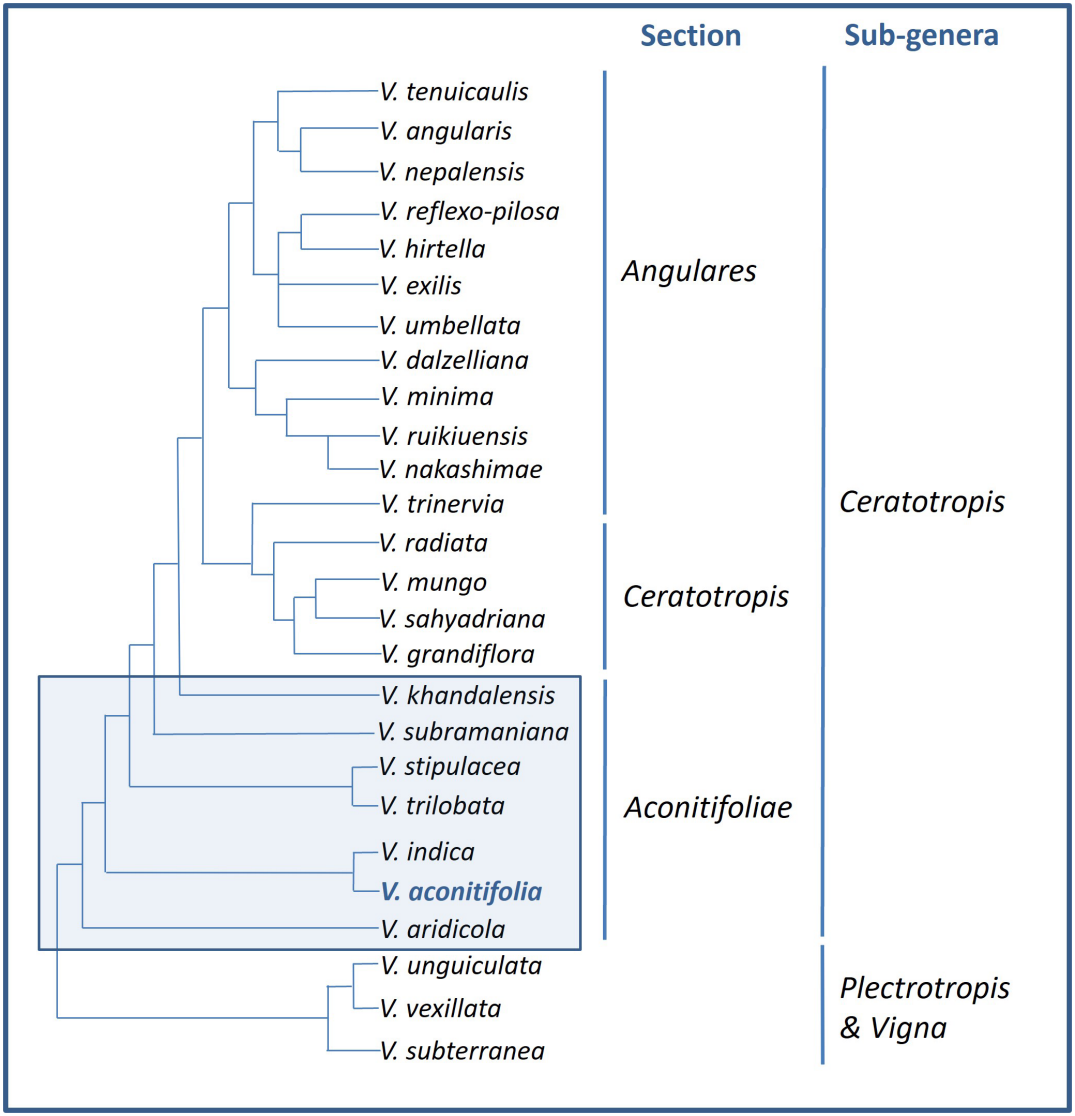
Taxonomic relationships among moth bean and other species of the genus *Vigna* [Source: Adapted from Takahashi et al. (2016)].

The wild, conspecific forms as well as cultigens of moth bean are not recognized (Maréchal, 1978). The precursor of moth bean is thought to be *Phaseolus trilobata* (L.) (syn. *Phaseolus trilobus*), which is a wild species endemic to India, with both being diploid and having chromosome number 2n=22 (Darlington and Wylie, 1955; Biswas and Dana, 1976a). *Phaseolus trilobata* and *Phaseolus aconitifolia* were considered to be equivalent by some while others believed *Phaseolus trilobata* to be the wild form of *Phaseolus aconitifolia*, however, studies have established both of them to be different (Sampson, 1936; Whyte et al., 1953; Klozová, 1965; West and Garber, 1967). Similarly, *Vigna aconitifolia* was classified with *Vigna radiata* and *Vigna mungo*, nonetheless, RAPD analysis to study genetic variability showed interspecific differences among them (Dana, 1980; Kaga et al., 1996). Takahashi et al. (2016) have performed molecular phylogenetic analysis of *Vigna* species using rDNA-ITS and *atpB-rbcL* sequences which divided the *Aconitifoliae* section into various branches, of which *V. aconitifolia* is one (**Figure 4**).

## 4 Nutritional profile

Moth bean is primarily consumed by people in the arid and semi-arid regions of South Asian countries, particularly India (Adsule, 1996; Takahashi et al., 2016). Ripe seeds are often consumed whole or split and can be cooked, roasted or fried. In India, a popular use of moth bean seeds is in the form of ‘*dal*’ (bean stew) or ‘*bhujia*’ (crispy snack), in addition to being used in the preparation of *papad, mangori, rabri and vada* (moth bean fritters) (Senthilkumar and Ngadi, 2020). The seeds are also ground into flour and often mixed with other flours to make unleavened bread (Brink and Jansen, 2006). Sprouted as well as cooked seeds are a popular breakfast dish while fried, split moth bean can be readily consumed (Medhe et al., 2019). Boiled immature green pods are consumed as a vegetable. As a medical use, the seeds are used to treat fevers. The roots are found to be narcotic (Brink and Jansen, 2006). Other pharmacological properties of Moth bean include anti-hypertensive, anti-oxidant, anti-cancerous, anti-bacterial, diuretic and hypocholesteromic activities (Adsule, 1996; Ma et al., 2010; Saravanan and Ignacimuthu, 2015; Panicker and Hamdule, 2021; Verma et al., 2021). Moth bean also contributes indirectly to human nutrition by being used as fodder for livestock.

In terms of the nutritional aspects of moth bean, it is considered as a good and inexpensive source of protein in cereal-based vegetarian diets among the people of developing nations. The protein content in moth bean seeds ranges from 20 to 24% (Gopalan et al., 1989; Adsule, 1996; Longvah et al., 2017). It is rich in the essential amino acids, lysine (6.63 g/100 g protein) and leucine (7.85 g/100 g) that are deficient in cereals and thus complements the amino acid profile of cereals. Among the total soluble protein present in moth, the glutelin fraction was found to be the highest at 63.93 g/100 g of total soluble protein, followed by glutelin, albumin and prolamin (Sathe and Venkatachalam, 2007). Additionally, the total carbohydrate content in moth bean was found to range from 52 to 68%, the total fat ranged from 1.1 to 3.9% while the crude fiber content ranged from 3.9 to 4.5%. Moth bean is also found to be rich in minerals such as calcium, magnesium, iron, phosphorus and potassium as well as vitamins like niacin, thiamine and riboflavin (Longvah et al., 2017; USDA FoodData Central, 2019). A comparison of the nutritional profile of moth bean with other pulses is illustrated in **Table 1**.

**Table 1:** Comparative analysis of the nutritional composition of moth bean with other pulses.

Composition	Chickpea (*Cicer arietinum*)	Lentil (*Lens culinaris)*	Kidney bean (*Phaseolus vulgaris*)	Dry peas (*Pisum sativum*)	Moth Bean (*Vigna aconitifolia*)	Black gram (*Vigna mungo*)	Green gram (*Vigna radiata*)	Bambara groundnut (*Vigna subterranea*)	Rice bean (*Vigna umbellata*)
**Energy (KJ)**	1201 ± 9	1251 ± 23	1252 ± 14	1269 ± 13	1291 ± 16	1219 ± 5	1229 ± 10	1514.78^a^	1265
**Carbohydrates (g)**	39.56 ± 0.16	48.47 ± 1.12	48.61 ± 0.65	48.93 ± 0.45	52.09 ± 0.96	43.99 ± 0.76	46.13 ± 0.64	55 ± 0.50^a^	51.26
**Protein (g)**	18.77 ± 0.42	22.49 ± 0.58	19.91 ± 1.44	20.43 ± 0.79	19.75 ± 0.38	21.97 ± 0.63	22.53 ± 0.43	22.46 ± 0.02^a^	19.97
**Fat (g)**	5.11 ± 0.11	0.64 ± 0.02	1.77 ± 0.04	1.89 ± 0.06	1.76 ± 0.09	1.58 ± 0.06	1.14 ± 0.17	5.80 ± 0.02^a^	0.74
**Moisture (g)**	8.56 ± 0.37	9.20 ± 0.77	9.87 ± 0.30	9.33 ± 0.61	8.14 ± 0.49	8.70 ± 0.33	9.95 ± 0.42	9.82 ± 0.02^a^	11.12
**Total dietary fiber (g)**	25.22 ± 0.39	16.82 ± 1.30	16.57 ± 0.63	17.01 ± 0.63	15.12 ± 0.49	20.41 ± 0.06	17.04 ± 0.38	4.50 ± 0.01^a^	13.37
Minerals and trace elements									
**Iron (mg)**	6.78 ± 0.75	7.57 ± 0.67	6.13 ± 0.77	5.09 ± 0.45	7.90 ± 0.1 7	5.97 ± 0.56	4.89 ± 0.46	18.51 ± 0.10^a^	4.76
**Magnesium (mg)**	160 ± 17.5	101 ± 13.9	173 ± 9.7	123 ± 8.1	205 ± 13.5	190 ± 19.1	198 ± 39.2	347.15 ± 0.01^a^	201
**Manganese (mg)**	2.17 ± 0.33	1.55 ± 0.26	1.24 ± 0.11	1.08 ± 0.09	1.07 ± 0.02	1.83 ± 0.34	1.05 ± 0.08	10.46 ± 0.05^a^	1.68
**Phosphorus (mg)**	267 ± 21.9	274 ± 27.4	409 ± 32.4	334 ± 18.3	362 ± 31.7	345 ± 36.5	353 ± 33.6	738.04 ± 0.15^a^	270
**Potassium (mg)**	935 ± 37.9	756 ± 63.6	1324 ± 195	922 ± 67.4	1356 ± 53.2 1	1093 ± 24.5	1177 ± 74.3	1702.10 ± 0.50^a^	1196
**Sodium (mg)**	26.56 ± 10.12	11.20 ± 0.08	10.45 ± 0.05	23.40 ± 0.07	26.34 ± 0.10	26.80 ± 3.77	12.48 ± 0.07	135.30 ± 0.05^a^	10.62
**Calcium (mg)**	150 ± 18.3	76.13 ± 7.23	126 ± 8.1	75.11 ± 13.93	154 ± 17.0	86.18 ± 8.99	92.43 ± 10.68	256.56 ± 0.05^a^	200
**Zinc (mg)**	3.37 ± 0.26	3.60 ± 0.23	2.69 ± 0.34	3.10 ± 0.14	1.92 ± 0.08	3.05 ± 0.24	2.67 ± 0.13	1.9 ± 0.1^b^	2.29
Vitamins									
**Thiamine (mg)**	0.37 ± 0.40	0.40 ± 0.073	0.30 ± 0.020	0.56 ± 0.049	0.45 ± 0.070	0.32 ± 0.024	0.45 ± 0.027	0.28^c^	0.46
**Riboflavin (mg)**	0.24 ± 0.011	0.22 ± 0.026	0.19 ± 0.018	0.16 ± 0.013	0.09 ± 0.005	0.11 ± 0.08	0.27 ± 0.011	0.12^c^	0.14
**Niacin (mg)**	2.10 ± 0.06	2.54 ± 0.12	2.42 ± 0.15	2.69 ± 0.15	1.87 ± 0.08	1.85 ± 0.13	2.16 ± 0.13	2.1^c^	2.32
**Biotin (µg)**	0.93 ± 0.07	1.74 ± 0.16	0.77 ± 0.18	0.53 ± 0.12	2.12 ± 0.21	1.28 ± 0.18	1.35 ± 0.16	–	2.65
**Total folic acid (µg)**	233 ± 12.9	132 ± 6.7	316 ± 20.1	110 ± 9.3	349 ± 10.8	134 ± 14.2	145 ± 5.4	–	122
**Vitamin E (mg)**	1.72 ± 0.07	0.19 ± 0.02	0.23 ± 0.01	0.32 ± 0.02	0.79 ± 0.05	0.23 ± 0.02	0.33 ± 0.02	8.22 ± 1.29^d^	1.06

(Source: Longvah et al. (2017); Oyeleke et al. (2012) ^a^; N’Dri Yao et al. (2015) ^b^; Hardy (2016) ^c^; Baptista et al. (2017) ^d^).

Moth bean, like other pulses, also contains flatulence-producing oligosaccharides such as raffinose, stachyose and verbascose. Also, like other legumes, it contains anti-nutritional and phytonutrient factors that affect the nutrient bioavailability as well as palatability and digestibility of moth bean. These factors include trypsin inhibitors, phytic acid, saponins and phyto-haemagglutinins (lectins). Trypsin and chymotrypsin inhibitors have been involved in reducing protein digestibility and thereafter in pancreatic hypertrophy (Liener, 1976). Phytic acid interferes with the bioavailability and absorption of minerals in the body and inhibits proteolytic enzymes and amylases while saponins are gastric irritants (Davies and Nightingale, 1975; Erdman, 1979; Singh and Krikorian, 1982; Deshpande and Cheryan, 1984; Pugalenthi et al., 2005; Bouchenak and Lamri-Senhadji, 2013). However, food preparation techniques, such as soaking, sprouting, boiling, pressure cooking as well as fermentation of moth, aid in improving the taste as well as the bioavailability of nutrients as they deactivate these anti-nutritional factors besides allowing the digestion and assimilation of its starch and protein (Khokhar and Chauhan, 1986; Kigel, 1999; Xu and Chang, 2008; Deshmukh and Pawar, 2020).

Regardless of being nutritionally dense, the present consumption levels of moth bean are rather low which can be attributed to its underutilized status. While there are indirect evidences that support its role in reduction of diseases, they are more based on its nutritional composition, therefore, focused research on the clarification of the health effects of moth bean should be undertaken.

## 5 Crop improvement

### 5.1 Plant genetic resources (PGR): status and trait discovery

**Figure 5:**
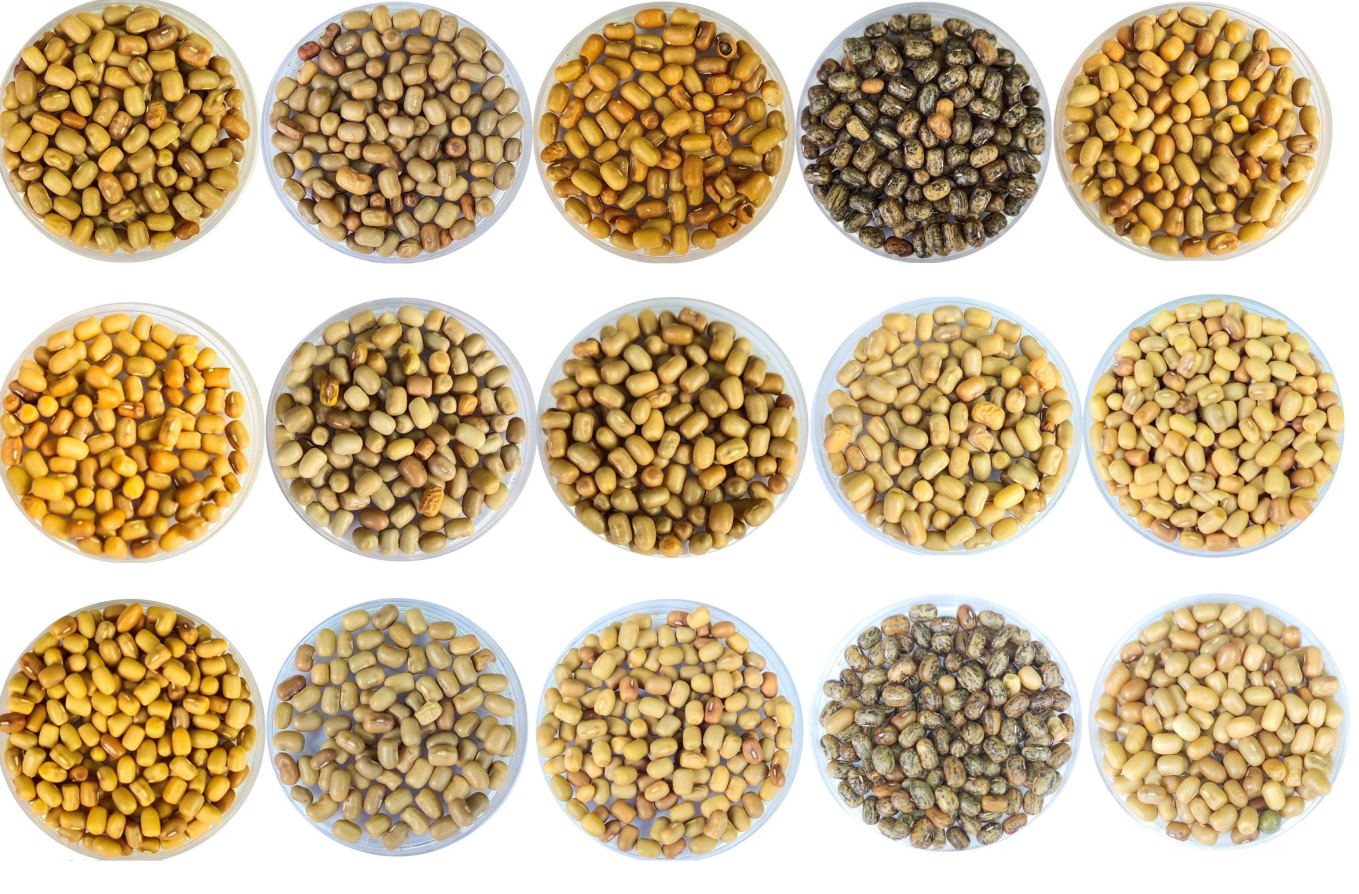
Variability in the shape, size and color of *Vigna aconitifolia* seeds conserved in the Indian National Gene bank, New Delhi.

Major moth bean *ex-situ* collections are maintained by the Indian National Gene bank, ICAR-National Bureau of Plant Genetic Resources, New Delhi, India. In India, systematic exploration and collection of the germplasm work commenced as back early as the 1940s, with the establishment of the Plant Introduction unit in the Division of Botany, IARI (Indian Agricultural Research Institute), New Delhi. Intensive collection efforts, carried out by National Bureau of Plant Genetic Resources, from 1971 till date, have resulted in the assembly of 3422 accessions of moth bean. These accessions include primitive cultivars/landraces, primarily from the states of Rajasthan, Gujarat, Maharashtra, Karnataka, western Uttar Pradesh, Punjab, Haryana and Madhya Pradesh. Variations in growth habit, leaf location as well as pod and seed color were observed in major collections of *V. aconitifolia*. The collections made from Rajasthan and Gujarat have turned out to be more promising and are currently being utilized in extensive characterization and evaluation programmes, to identify superior genotypes (Bisht and Singh, 2013). Besides germplasm collections from within the country, NBPGR further plays a crucial role in the augmentation of the germplasm from other countries as well. 41 accessions of moth bean have been introduced from Sri Lanka, USA (19), Mexico, USSR, Thailand (5) and others (14). Other small collections are being held by institutions worldwide are University of Georgia, USDA (56 accessions), VIR (48), Australian Grain Gene bank (35), AVRDC-The World Vegetable Centre (26), CIAT, Columbia (8) and Leibniz Institute of Plant Genetics and Crop Plant Research (6) (**Table 2**; **Figure 3**). Moth bean has orthodox seeds that can be dried and stored for a longer period with minimum to no loss of viability. A limited range of germplasm accessions can also be found in countries such as Bangladesh, Belgium and Kenya. Active collections are being maintained at NBPGR, Regional Station, Jodhpur (Rajasthan). The working collections of a range of *Vigna* species are also maintained at the Indian Institute of Pulses Research (IIPR), Kanpur, India and its coordinating centers (Asthana, 1998). More than 2000 accessions of moth bean have been characterized and evaluated at NBPGR, Regional Station, Jodhpur (Singh et al., 2001). Yield and other growth characteristics have shown a wide range of variation, as demonstrated in **Table 3**. Nodulation and nitrogenase activity as well as resistance to insect pests and diseases have shown extensive varietal divergence in moth bean (Rao et al., 1984; Dabi and Gour, 1988). Quality characters have also demonstrated a substantial amount of variability (**Figure 5**).

**Table 2:** Moth bean germplasm holdings at some key centers worldwide.

Countries	Institutes/Centers	Accessions
India	National Bureau of Plant Genetic Resources, Jodhpur	3422
Pakistan	National Agricultural Research Centre (NARC), Islamabad	66
Japan	NARO (National Agriculture and Food Research Organization)	43
Kenya	Kenya National Gene Bank of Kenya, Crop Plant Genetic Resources	50
Russian Federation	N.I. Vavilov Research Institute of Plant Industry Russian VIR, St Petersburg	64
Taiwan	World Vegetable Centre, Taiwan AVRDC, Taiwan	26
USA	Plant Genetic Resources Conservation Unit, Southern Regional Plant Introduction Station, USDA-ARS, Griffin, GA	58
Australia	Australian Grains Genebank	36
Australia	Australian Pastures Genebank, Australia	28
Ukraine	Ustymivka Experimental Station of Plant Production, Ukraine	35
Belgium	Botanic Garden Meise, Belgium	7
Colombia	Centro Internacional de Agricultura Tropical, Colombia	8
Germany	Gene bank, Leibniz Institute of Plant Genetics and Crop Plant Research, Germany	7
Brazil	Embrapa Recursos Genéticos e Biotecnologia, Brazil	7

(Source: Agrawal et al. (2019); Zahoor (2007), www.genesys-pgr.org, www.gene.affrc.go.jp).

**Table 3:** A comparative account of agronomic traits of moth bean.

Traits	Sihag et al., 2004	Singh et al., 2006	Kohakade et al., 2017	Sahoo et al., 2019	Overall range
**Days to 50% flowering**	37.85 (29.5-47.5)	63.73 (32.0–84.0)	84.93 (43.7-106.7)	46.29 (36.3-50.7)	29.50-106.67
**Day to 80% maturity**	73.78 (54.5-89.5)	82.84 (57.0-105.0)	135.09 (90.3-158.7)	72.57 (61.7-91.0)	54.50-158.67
**Plant height (cm)**	51.35 (37.5-94.3)	26.45 (11.7–49.3)	107.44 (11.6-153.1)	32.07 (19.5-40.4)	11.63-153.13
**Primary branches/plant**	–	6.09 (1.6-12.0)	5.01 (2.3-8.8)	04.37 (3.2-6.0)	1.58-12.00
**Clusters per plant**	22.23 (4.4-55.1)	20.98 (9.0-73.7)	35.08 (14.8-58.8)	–	4.40-73.67
**Pods per plant**	62.57 (20.0-129.0)	49.52 (24.8-128.3)	66.05 (20.8-145.2)	98.66 (65.3-148.8)	20.00-148.83
**Pod length (cm)**	3.51 (2.4-4.0)	3.95 (2.2–5.3)	3.37 (2.9-3.8)	3.99 (3.3-5.5)	2.20-5.51
**Seeds per pod**	5.74 (4.85-7.0)	6.75 (2.0–10.0)	5.67 (3.2-6.9)	4.96 (3.6-6.9)	2.00-10.00
**100-seed weight (g)**	2.39 (1.6-3.2)	2.90 (1.5–4.6)	2.32 (1.5-2.9)	2.60 (1.7-3.3)	1.50-4.60
**Yield/plant (g)**	5.74 (0.9-12.1)	6.17 (1.6–19.1)	6.94 (1.8-20.6)	11.41 (4.3-20.5)	0.94-20.64

### 5.2 Conventional breeding

Initially, moth bean breeders focused mainly on the maintenance of crop genetic resources, germplasm evaluation, and genetic diversity assessment (Cullis and Kunert, 2017). Owing to the difficulty in handling the tiny flowers that drop off during crossing, hybridization programmes were unsuccessful in minor legumes like moth bean resulting in lack in genetic studies. However, studies on germplasm evaluation resulted in the identification of promising genotypes in addition to providing information on diversity, heritability, and other genetic parameters as well as the correlation between traits and environmental influences that could be useful in expediting genetic gains (Vir and Singh, 2015; Kohakade et al., 2017; Sahoo et al., 2019; Pal et al., 2020; Chaudhary et al., 2021). A few promising accessions identified through germplasm evaluation, are IC36245, IC36555, IC36667, IC36577 and IC36604 (Vir and Singh, 2015). Likewise, Sihag et al. (2004) reported that in terms of the traits studied, MH 65, MH 34/66, MH 45, and MH 66 were the most promising genetic stocks, and their use in the moth bean varietal improvement programme could be propitious. Similarly, Kohakade et al. (2017) identified five promising genotypes (DHMB-32, DHMB-26, DHMB-31, DHMB-30 and DHMB-16) under the hybridization programme based on inter-cluster distances, cluster mean and per se performance. Systematic germplasm evaluation studies by various workers have identified valuable trait-specific accessions for moth bean improvement (**Table 4**). In moth bean breeding programmes, these accessions could be a valuable resource for improving moth bean resistance to major biotic and abiotic stresses and for enhancement of grain yield. Variety development programme in moth bean was probably initiated in the late 1960 or early 1970s (Singh and Mann, 1976). Traditional germplasm-selected varieties were of spreading types that covered the ground like a mat, grew slowly, and matured in 100-120 days. These varieties (Type-I, Type-3, MG-l, Baleswar-12, and so on) were introduced prior to 1970. However, under ideal conditions, these fodder types barely give about 200-300 kg/ha of yield (Kumar, 2002). Further selections from the germplasm resulted in identification of high yielding varieties with long and medium duration (Maru moth) maturity before the inception of AICRP on Arid legumes in 1992 (**Table 5**). Work on arid legumes *viz.*, cluster bean, moth bean, cowpea and horse gram started in 1992. High-yielding varieties that mature in 75-90 days have thus been developed as a result of continuous, sincere, and deliberate efforts to improve this crop’s genetics and yield. As a result, the newer cultivars (Jadia, Jawala, IPCMO-912, and CAZRI moth-1) could produce higher yield while also withstanding the extreme heat and limiting soil moisture conditions. These varieties were also better suited to areas with high levels of rain (250- 350 mm) (Kumar, 2002). In 2003, CAZRI moth-2 (CZM-2) was developed by Central Arid Zone Research Institute, which was the first variety to be released through hybridization and has gained wide popularity in recent years owing to its high yield potential and considerable podding (Solanki et al., 2018). In addition to these, its introduction to the marginal irrigated farming system, development of early maturing, photosynthetically efficient plants, breed of input responsive genotypes, development of varieties suitable for inter/mixed cropping system, identification of ‘dual types’ (high grain as well as fodder yielder) and development of varieties resistant to biotic and abiotic factors are some of the priority areas which should be taken care of in future genetic improvement programmes on moth bean.

**Table 4:** Potential trait-specific accessions identified in germplasm for utilization in moth bean improvement.

Traits	Materials used	Trait attributes considered	Promising entries	References
Agronomic traits	49 genotypes	Pods per plant, seeds per pod, pod length, hundred seed weight (g), biological yield, harvest index, and seed yield/plant	IC370469, GP387, IC415116, IC310670, IC329040, RMO225, RMO-257, IC983	Pal et al., 2020
Early maturity	Mutant of moth bean variety RMO-40	Phenological traits like days to initial flowering, days to 50% flowering, days to maturity,	IC432859	Bisht and Singh, 2013
	62 genotypes	Pod length and seeds/pods	MH 65, MH 34, MH 66, and MH 45	Sihag et al., 2004
Heat tolerance	10 germplasm lines & varieties	Relative water content (RWC), membrane stability index (MSI), and proline, protein, and chlorophyll (Chl)	MO 40, Jadia, IC36157, Jwala, and Maru moth	Tiwari et al., 2018
Drought tolerance	Mutant of moth bean variety Jadia	Relative water content (RWC), membrane stability index (MSI), and proline, protein, and chlorophyll (Chl)	IC296803	Bisht and Singh, 2013
	32 accessions	Plant height, root length, branches plant dry mass, root dry mass, chlorophyll & relative water content	IC129177, IC103016, IC415139, IC415155 and IC36157	Malambane and Bhatt, 2014
YMV resistance	204 accessions	Disease intensity (%), Disease incidence (%) & Plant Disease Index (%)	PLMO 12, IC36096, IC415152, IC129177, IC129177,	Meghwal et al., 2015
	11 grain type & 10 fodder type germplasm	YMV disease incidence was recorded on a single-plant basis	013393-C, DMB-118-E	Yaqoob et al., 2015
Leaf crinkle virusresistance	44 accessions	Leaf crinkle disease scoring on a single plant basis	IC39786	Vir and Singh, 2015
Phytochemicals	25 accessions	Protease inhibitors, phytic acid, radical scavenging activity, and tannins	IC39784, IC8851, IC39711 and IC39774	Gupta et al., 2016
	60 accessions	Comprehensive biochemical profiling includes higher contents of magnesium and iron	IC36361, IC-311448, IC415152 and RMO-257	Bhadkaria et al., 2021

**Table 5:** List of moth bean varieties developed through conventional breeding approaches in India.

Variety	Maturity (Days)	Grain yield (Kg/ha)	Salient features
Type-1	120	200-300	It was selected from a local collection, medium-sized brownish-red seed with an average forage production of 10-14 q per ha
Type3	120	370-375	Forage type, selected from a local collection, produces 22-25 q/ha of green forage, has narrowly lobed leaves, and has a canopy that extends outward and trails horizontally.
MG-l	110-115	350·450	Selected from a local collection, extremely vulnerable to YMV, plants are taller (45-55 cm), harvest Index is poor (10-12 percent)
Baleshwar-12	110-115	400-475	Selected from a local collection; plants are taller; green fodder yields 15–17 q/ha; seeds are brown and medium size; extremely vulnerable to YMV.
Jadia	85-90	450-500	Selected from a local collection, has a spreading growth habit, dark brown, medium bold seeds (l00-seed weight of 2.5-2.5g), is prone to yellow mosaic virus, and produces 10–12 q/ha of green fodder production.
Jawala	80-90	500-550	Selected from a local collection, resistant to YMV, and with an average fodder yield of 17-18 q/ha.
Maru moth	80-85	500-550	Selected from the local collection, this variety is a semi-spreading type that performs well in planting situations that are less affected by the *Cercospora* leaf spot disease. It is also suitable for intercropping.
IPCMO-800	80-85	450-500	Selected from a local collection; vining type; has broad, deeply lobed leaves; seed protein is 22–24 percent; harvest index is 20–25.
IPCMO 880	80-85	450-500	Pure line selection from a local collection in the Jhunjhunu district; matures in 90-100 days; produces 3-4 pods per cluster with medium to bold seed (2.8-3.1 g/100 seeds); seed yield: 4-5 q/ha.
IPCMO-912	75-85	400-500	This variety shows field tolerance to YMV and Bacterial blight, narrow leaflets.
CAZRI Moth-1	72-75	400-500	It is semi-spreading and dual-purpose variety responding to inputs, Field resistance to YMV, bold grain with 25% protein
CAZRI Moth-2	75-85	1000-1200	This is the first variety produced through hybridization (RMO-40 x Jadia); erect plant habit and profuse podding.

(Source: Kumar (2002); Solanki et al. (2018)).

### 5.3 Interspecific hybridization

In India and other Asian countries, *Vigna* species constitute an economically significant group of 10 cultivated crops (domesticated species) and more than 100 wild species (Schrire et al., 2005; Bisht and Singh, 2013; Takahashi et al., 2016). Moth bean is the most interesting of the Asian Vigna species that have been domesticated as it is highly resistant to drought and heat (Tomooka et al., 2014). The wild ancestor of the moth bean hasn’t been precisely described, but it is said to have occurred in India (Arora and Nayar, 1984). Understanding the genetic basis of crop domestication aids in the identification of beneficial genes (or genes) in wild relatives of cultivated crops that can be used in plant breeding. In addition, as a reliable strategy for expanding a crop species’ genetic base, interspecific hybridization has successfully introduced beneficial traits from wild relatives into closely related crops (Singh, 1990). Varying degrees of success in interspecific hybridization of *Vigna* has been reported (Chen et al., 1983; Singh, 1990; Pandiyan et al., 2012; Tomooka et al., 2014; Basavaraja et al., 2019). Cross-compatibility between *V. aconitifolia* and other relative species hasn’t been studied much, but it has been crossed as a seed parent with *V. trilobata*, and *V. trilobata* has been crossed with success, as a pollen parent, with *V. mungo*, *V. radiata*, and *V. aconitifolia*, but the reciprocal hasn’t reportedly worked (Bisht et al., 2005). The interspecific hybrids between *V. aconitifolia* and *V. trilobata* produced viable seeds. The F_1_ hybrids had 5.7% pollen fertility, but complete seed sterility. Colchicine-induced amphiploids had 89.7% pollen fertility and such plants had fairly regular meiosis, suggesting the possibility of such crosses. Hybrid sterility is segregational in nature (Biswas and Dana, 1976b).

### 5.4 Tissue culture approach

Through interspecific hybridization, efforts have been made in the recent past to develop disease-resistant as well as high-yielding varieties of moth bean. However, interspecific cross incompatibility and hybrid sterility are some significant hurdles for moth bean variety development programme. Consequently, it is conceivable that moth bean quality and yield can be improved by using a combination of tissue culture techniques and traditional breeding methods. Tissue culture is a potent method that enables scientists to cultivate and manipulate plants in a sterile environment. It is an advantageous method for plant breeding. Therefore, cell and tissue culture application to moth bean assumes a special significance. However, the lack of or low rate of plant differentiation from cultured cells is one of the major obstacles towards the utilization of cell and tissue culture techniques in grain legume breeding programmes (Bajaj and Gosal, 1981; Gresshoff and Mohapatra, 1982; Mroginski and Kartha, 1984; Saxena, 2004). Several studies have reported that many legumes have now been successfully cultured and regenerated as whole plants. Bhargava and Chandra (1983) initiated callus cultures of two cultivars of *Vigna aconitifolia* (moth bean) (IPCM0-926, RDM-120) and studied their growth and differentiation. Likewise, Godbole et al. (1984) optimized the cultural conditions for whole moth bean plant regeneration from explant shoot apices and cotyledons, callus cultures derived from explant shoot apices, and field transfer of rooted plantlets. As a practical issue, the development of shoots and subsequent plantlets from excised roots is of interest because it provides a new source of somaclonal variation. Gill and Eapen (1986) attempted to develop cell and tissue cultures in moth bean, whereby a successful attempt was made in moth bean plant regeneration from hypocotyl protoplasts. Eapen and Gill (1986) were able to be regenerate plants from root explants of moth bean by culturing on Murashiage and Skoog’s basal medium without any phytohormone, but the addition of cytokinins increased the frequency of plant regeneration.

Protoplasts, on the other hand, are also an excellent source of inducing variation in *Vigna*. Protoplasts from this important group of economic crops have been isolated and regenerated into cell colonies and callus tissues. Callus cultures derived from shoot apices of moth bean provided a high yield of viable moth bean protoplasts as a starting point for the development of an efficient and reproducible moth bean protoplast isolation and regeneration method (Krishnamurthy et al., 1984). Similarly, Arya et al. (1990) regenerated plants from leaves’ protoplasts of *Vigna aconitifolia* ‘Jadia’ and reported that protoplasts from highly homozygous legumes are of application in moth bean improvement. Kumar et al. (1988) demonstrated successful plant regeneration from cell suspension cultures lines of *Vigna aconitifolia* in L-6 medium containing 44.5 M 2,4-D, which resulted in the highest growth rate. Till date, no systematic study has been reported in moth bean on the effects of cultivar, basal medium, different medium combinations (Murashiage and Skoog medium (MS), 2,4-D & 6-Benzylaminopurine (BAP) and interactions on callus induction, callus propagation and subsequent plant regeneration (Bhargava and Chandra, 1983; Bhargava and Chandra, 1989). Furthermore, Jangid et al. (2010) studied factors affecting callus induction in moth bean and found significant differences, for both callus initiation days and callus fresh weight, between the varieties, explants, medium combinations, and their two- and three-way interactions while also reporting that MS medium supplemented with 1.0 mg-1, 2-D was the best medium for maintaining callus in all of the tested types. There have been numerous attempts made to develop moth bean regeneration protocols *in vitro* (Arya et al., 1990; Chandra and Pental, 2003; Kaviraj et al., 2006). When tested on Indian moth varieties, the *in vitro* regeneration techniques developed by these researchers failed to deliver the expected outcomes. Hence, Choudhary et al. (2009) developed an effective method for regenerating moth bean using somatic embryos and *in vitro*-grown plantlets that resulted in well-formed roots being successfully hardened and established in soil. Recently, regeneration potential, gene expression, and genetic stability were found to be affected by heat treatment in moth bean (Jangid et al., 2010). Additionally, the response of tissue to callus formation was studied in explants that had been heated at 37, 42, or 47°C for 10 minutes (Sharma et al., 2018). The results showed that most of the heat treatments slowed down regeneration, and a few polypeptides in the protein profile of the callus were both up-regulated and down-regulated by the heat treatments. Understanding the mechanism of flowering regulation in *in-vitro* cultures is practical and theoretically relevant. The effect of growth regulators such as abscisic acid and proline on *in vitro* flowering manipulation in moth bean has been investigated by Saxena et al., 2008. On the contrary, moth bean presents an efficient system to be used for *in vitro* mutagenesis because it exhibits an efficient regenerability from various explants and bear seeds under *in vitro* conditions (Saxena et al., 2006). Likewise, Narayan et al. (2015) demonstrated that gamma radiation exposure increased the number of shoot bud primordia and the number of shoots per explant in callus generated from primary leaves of seedlings of moth bean seeds. Such explants also resulted in a higher number of flowering plantlets under *in vitro* growth conditions. Considerable success has been achieved in moth bean *in vitro* culture plant regeneration, and this progress offers an alternative option, over conventional breeding or interspecific hybridization, for the improvement of moth bean.

### 5.5 Mutation breeding

Mutation is a highly effective technique to generate the desired variation in crop plants. It has been extensively used in many of the world’s most important crops, including wheat, rice, pulses, millets, and oilseeds, and several original and review studies show its effectiveness (Khadke and Kothekar, 2011; Raina et al., 2016; Jegadeesan and Reddy, 2018; Pandit et al., 2021). Work on the varietal improvement of moth bean crop is minimal; a mutation breeding programme in moth bean was taken up in 1983, to isolating mutants with high yield potential, early maturing and at the same time possessing tolerance to drought conditions. Henry and Daulay (1983) researchers examined the performance of induced mutants in moth bean, treating seeds of the Jadia variety with aqueous solutions of EMS at doses ranging from 0 to 3 percent. In the M_2_ generation, they found 25 mutants with higher pods than their parents. The mutants were further carried forward up to M_5_ and M_6_ generation and tested under dry land conditions for yield performance, along with other high-yielding varieties. On the contrary, to improve arid legumes, a mutation breeding technique utilizing chemical and physical mutagens was undertaken, (Ramkrishna et al., 2008; Jain et al., 2013). The most commonly used mutagens, namely, Ethyl methane sulphoanate (EMS), Methyl methane sulphonate (MMS), Sodium azide (SA) and Hydroxylamine (HA) were used in moth mutation breeding and the effectiveness of the mutagens was tested on three different bean crops. Moth bean genotypes had the highest efficiency, followed by cluster bean, while mung bean genotypes had the lowest efficiency (Sharma and Kakani, 2002; Ramkrishna et al., 2008). Four moth bean varieties, RMO-40, RMO-257, Jwala and CZM-1, were tested for their response to EMS, MMS, SA, and HA, induced biological damage, polygenic variability, and comparative mutagenic effectiveness and efficiency. This was performed to better understand how different moth bean varieties respond to different types and treatments of chemical mutagens. Subsequently, the mutagenic progenies, concerning yield and yield attributes in M_2_ and M_3_ generation, were evaluated. Treatment efficacy declined in the following order: MMS > SA> EMS > HA. The moth bean variety, RMO-40, was shown to have the maximum mutagenesis effectiveness with a concentration of 1.0 mM Sodium azide. In M_3_ generation, 15 progenies showed higher seed yield with superior magnitude of yield contributing traits than the best check, RMO-225 (Jain et al., 2013).

Many national and state agricultural universities, including Bhabha Atomic Research Centre (BARC), Mumbai (India), have been doing intensive mutation research since decades by employing X-rays, gamma rays, fast and thermal neutrons in order to generate genetic variability in these arid legume crops (D’Souza et al., 2009). As a result, research on induced mutations in oilseeds and legumes has remained at the leading edge of Indian agricultural research for developing popular varieties with higher productivity in these crops. Khadke and Kothekar (2018) studied the effect of EMS and SA on moth bean trypsin inhibitor content. On electrophoresis, various viable and micro mutants of moth bean were found to have between three and seven iso-inhibitors of trypsin. Some viable mutants had considerable differences in their TI profiles when compared to the control. The TI content of these mutants reduced by 25 to 45 percent, and the seed protein content of micro mutants and viable mutants differed significantly. In 1994, the release of the first early maturing mutant variety, RMO-40, sparked an interest in mutagenesis, leading to the development of a series of short-duration varieties, including RMO-257, RMO-225, RMO-435, RMO-423, and RMO-2251 (**Table 6**). All of these cultivars were early maturing (60-67 days), exhibited completely transformed plant types, were semi-erect to erect, and had synchronous maturation. Due to short growing seasons, these varieties could successfully fend off drought problems and circumvent devastating diseases like YMD (yellow mosaic disease) and *Cercospora* leaf spot. Such varieties are more suited to low rainfall (200-250 mm) and shorter growing season. Varieties developed through mutation breeding could be direct mutants with good agro-morphological traits or the resultants of hybridization with mutants for mutant trait introgression in more adaptable and elite genetic backgrounds Pulses with narrow genetic bases have enormous potential for increasing genetic variability through a combination of mutation breeding and conventional breeding approaches. This could lead to the development of elite varieties that are suitable for various agro-climatic zones, thereby improving pulses production subsequently leading to nutritional security.

**Table 6:** Popular high yielding moth bean mutant varieties under cultivation in India.

Mutant variety	Maturity (days)	Yield (kg/ha)	Special features	References
RMO-40 (Rajasthan Moth 40)	62-65	600 to 900	This is an extra early maturing, escapes drought, short stature, and non-spreading variety with synchronous maturity.	Sharma and Kakani, 2002
RMO-225 (Maru vardan)	64-67	600 to 750	This variety has a short duration and a high level of resistance to the yellow mosaic virus.	Kumar, 2005
RMO-435	67-70	600 to 800	This variety is erect with medium duration, has good yield potential, leaves green colored, mutant from RMO-40and has fodder value. Therefore, it is suitable for all moth-growing areas of the country.	Kumar, 2005
RMO-257	65-67	500 to 550	This mutant from RMO-225 variety is utilized for both grain and fodder production. It is a variety that matures early and has a moderate level of resistance to bacterial blight and YMV. Its flowers are arranged in clusters and each one has a little petiole.	Jain et al., 2013; Sharma et al., 2015
RMO-423	67-70	550 to 600	The cultivar can be used for either seed or fodder cultivation. It can also resistant attacks from insects and the yellow mosaic virus.	Jain et al., 2013
RMB 25	67-70	600 to 700	It gives a higher seed (28.6%) and fodder yield (15.4%) than RMO-225 and RMO-257. The variety has field resistance to yellow mosaic virus and high protein content.	Sharma et al., 2015
RMO-2251 (Maru dhar)	63-67	600 to 650	It is a short duration variety with early maturity, erect plant type and average incidence of YMV.	Solanki et al., 2018
CAZRI Moth-3	62-64	550-750	Escapes YMV. Early maturing and erect. Heavy pod-bearing. Drought tolerant.	Solanki et al., 2018

## 6 Abiotic and biotic stress tolerance and adaptive traits in moth bean

### 6.1 Heat stress tolerance

Moth bean is a highly adaptable annual legume that thrives in arid and warm climates, making it one of the most temperature-resistant legumes in arid climates (Sachdeva et al., 2016). It has a deep and fast-penetrating root system, can thrive in open fields for up to 30-40 days, and can withstand temperatures of more than 45°C. By virtue of its adaptive features, whereby it can sustain harsh environmental conditions, moth bean is widely recognized as an arid legume. To mitigate heat stress, moth bean has developed morphological accommodations such prostrate development thereby shading the soil to reduce temperature, large leaves with dense canopy for transpirational cooling, higher biomass but poor partitioning, indeterminate growth, and a fast-penetrating tap root system (Sharma et al., 2014; Harsh et al., 2016). The crop appears to have a genetic buffer that allows it to quickly adapt to rapidly changing moisture conditions as well as deprived and hot environments. Temperature stress, contrarily, has been shown to speed up plant growth in moth bean begetting reduction in both vegetative and grain yields (Khadke et al., 2011; Sharma et al., 2014). Similar results were observed in a study based on the induction of thermo-tolerance for deciphering the efficaciousness of heat acclimation in nine different varieties of moth bean, which showed that all nine varieties could tolerate a sudden temperature rise of 42°C, and that the development of thermo-tolerance was linked to the induction of peroxidase (POX), ascorbic peroxidase (APOX), and catalase (CAT) enzymes (Sharma et al., 2014; Harsh et al., 2016). Likewise, the accumulation of total sugar and proline, as well as an increase in the activity of CAT, GPOX (glutathione peroxidase), and SOD (superoxide dismutase), was seen in thirty-seven genotypes of moth bean under heat stress in earlier investigations. (Harsh et al., 2016; Tiwari et al., 2018) and by that mutagenesis was suggested as an effective means of improving thermo-tolerance by altering the osmo-protectants and anti-oxidative enzymes that go along with it.

### 6.2 Drought tolerance

As a drought-tolerant crop, moth bean has had a long history of success in rain-fed arid environments. Pre-meditated and need-based efforts have been made for decades to increase the productivity and adaptability of this drought-resistant crop in harsher and more hostile conditions. There has been paucity in systematic research on the physiological basis of yield in rain-fed conditions and the mechanism for drought resistance in this crop, despite its economic importance. Physiological investigations of yielding ability in moth bean types under rain-fed conditions established that the crop is a poor seed producer despite producing enough dry matter. Leaf water potential, osmotic potential, and pressure potential were measured at regular intervals over a period of time. There were noticeable changes in transpiration amongst the different varieties (Srivastava and Soni, 1995). It is quite difficult to predict the outcome of crop production in arid and semi-arid areas. However, the choice of proper plant type and adopting an appropriate cropping system may provide yield stability in the region. The importance of the adoption of suitable genotypes and cropping systems has been emphasized by several investigators (Singh and Reddy, 1988; Lawn, 1989). Higher photosynthetic rates and more effective water use have been linked to the increased performance of mixed crops under low-rainfall conditions. Kathju et al. (2003) suggested that under both low and high rainfall situations, planting early and late genotypes in alternate rows at a 1:1 ratio is an ideal strategy for maintaining the production stability of moth bean. Priyanka et al. (2011) investigated the effect that a low water potential, caused by PEG (polyethylene glycol), has on growth, sugar content, and enzymes related to stress (such as catalase, GPOX). Additionally, SDS-PAGE (Sodium dodecyl-sulfate polyacrylamide gel electrophoresis) analysis was performed on seedlings of twelve moth bean genotypes and traits such as seed set and abscisic acid concentration in the pods as well as root traits have been proven to increase the tolerance of moth bean to drought (Kumar et al., 2019). Accordingly, Garg et al. (2001) reported that on rise in water stress in three moth bean genotypes (RMO-40, Maru moth, and CZM-32 E), net photosynthetic rate and starch and soluble protein content dropped besides a marked decline in the nitrate reductase activity of the plant. Discordantly, there was a build-up of reducing and soluble sugars as well as proline content at pre-flowering phase of the genotypes under study. A germplasm collection comprising of 32 genotypes was evaluated under both dry (terminal drought) and wet (rain-fed) environments. The results exhibited significant differences for genotypes, treatment (stress), and the GxS effects. Also, the differences in the amount of chlorophyll were significant for genotype and combination of genotype and stress treatment. The treatment effect showed a significant difference and led to the identification of two varieties (Maru moth and Jadia) and five accessions (IC 129177, IC 103016, IC 415139, IC 415155, and IC 36157) that were drought tolerant (Malambane and Bhatt, 2014). In another study, Sachdeva et al. (2016) reported that three cultivars (IC103016, IC36011 and IC36157) were more drought tolerant because they sustained greater RWC (Relative Water Content) and MSI (Membrane Stability Index) along with plant height, leaf area, seed weight and plant dry mass besides higher levels of proline accumulation. Recognized as having a primitive plant type, moth bean has evolved to survive but not for increased productivity. In this context, early partitioning, early maturation, and semi-erect to erect growth habit types may be preferred to the conventional sort. Consequently, such advantageous plant traits will not only lead to an increase in yield but also encourage the cultivation of this crop in new and unexplored areas.

### 6.3 Salinity tolerance

Regulation of proline biosynthesis as well as its degradation, uptake and transport are one of the key mechanisms of the plant defense system, which plays a compelling role in the survival of osmotically stressed plants (Stewart and Lee, 1981; Verma et al., 1992). The gene encoding the enzyme, ornithine delta-aminotransferase (delta-OAT) that is involved in proline biosynthesis pathway, was isolated from moth bean cDNA library by complementation of *Escherichia coli* proBA mutant auxotroph (Delauney et al., 1993). Zhu et al. (1998) transformed rice var. Nipponbare with P5SC gene obtained from moth bean and increased biomass in salt and water stress conditions. Similarly, when the rice cultivar, ADT 43, was transformed with Δ1-pyrroline-5-carboxylate synthetase (P5CS) gene obtained from moth bean and it was observed that under 200 mM NaCl, the transformed plants showed better plant growth and biomass production while the control plants died within 10 days (Karthikeyan et al., 2011). In another study, the overexpression of P5CS gene derived from moth bean in rice cultivar IR-50, showed tolerance to high salt (200 mM NaCl) conditions (Anoop and Gupta, 2003). Surekha et al. (2014) used *P5CSF129A*, a mutagenized P5CS gene obtained from moth bean, in combination with CaMV35S, a constitutive promoter, for the genetic transformation of *Cajanus cajan* to enhance proline accumulation and salt tolerance. Moreover, the P5CS gene from moth bean was transplanted into the hybrid larch (Larix x leptoeuropaea (Dengler)) to improve the productivity of Larch, a tree species that thrives under stresses like cold, salt, and frost, by which the transgenic tissues exhibited a thirtyfold increase in proline concentration (Gleeson et al., 2005).

### 6.4 YMV disease resistance

Biotic stresses such as Yellow Mosaic Disease (YMD) and the pod borer extend major production challenges for various *Vigna* crops. Like other *Vigna* crops, moth bean has not received the proper scientific attention towards management of biotic stresses, and among many, the crop requires particular consideration of YMV and Bacterial Leaf Spot disease. The *Mung bean yellow mosaic virus* (MYMV) is resistant to mung bean’s morphological and biochemical diversity, according to recent investigations. In terms of resistance, morphological characteristics such as leaf thickness and trichome density are varied. Leaf thickness was found to be higher in the MYMV-resistant genotypes compared to the highly sensitive ones. Similarly, high levels of trichome density were found in resistant genotypes, compared to low levels seen in the most susceptible ones (Patel et al., 2013; Mantesh et al., 2020). In another study, 204 moth bean lines were screened for resistance to the *Mung bean yellow mosaic virus* (MYMV). Thirteen of the 204 accessions studied showed resistance or tolerance to MYMV in the field (Meghwal et al., 2015). Stress tolerance genes must be identified which requires substantial information on the control of gene expression as well as the biochemical activity of specific proteins involved in the process of tolerance. According to Tiwari et al. (2018), the genes for stress-enzymes such as Catalase, Cyt P450 monooxygenase, heat shock proteins (HSP 90 and HSP 70), oxidoreductase, protein kinases, dehydration-responsive protein (DRP), universal stress proteins, and ferridoxin NADH oxidase were over-expressed in stressed samples. Additionally, expression profiling revealed ten transcripts to be up-regulated and 41 to be down-regulated, whereas 490 exhibited no significant change under moisture stress (Tiwari et al., 2018).

### 6.5 Insect resistance

Specific management measures to reduce yield losses due to insect pests, such as Jassids, white flies, pulse beetles, white grubs, and other storage pests is imperative *Callosobruchus chinensis* L. and *Callosobruchus maculatus* L. are major bruchid pests of most *Vigna* species, particularly cultivated ones. In light of this, bruchid resistance enhancement is prioritized in all *Vigna* crop breeding programmes. Plant breeders have long aimed to improve the bruchid resistance of cowpeas, mung beans, adzuki beans, and black gram. These *Vigna* crops have sources of bruchid resistance, but the resistant germplasm is limited. A wild moth bean accession, TN67, was recently discovered to be extremely resistant to *C. chinensis* (Somta et al., 2018). For *Vigna* crop breeding, wild moth bean’s resistance gene(s), including moth bean with *C. chinensis* resistance could be highly favorable (Somta et al., 2018).

## 7 Biotechnological interventions

### 7.1 Genetic linkage map and QTL mapping for agronomic traits

The work done in moth bean, with respect to breeding, is exiguous, therefore, mapping populations, core sets or trait specific reference sets are lacking in the crop. In addition, studies analyzing the genetic variability in *V. aconitifolia* germplasm, by way of molecular techniques like DNA markers, have been sparse (Sharma et al., 2021). Nevertheless, recently, there has been some progress. Despite having the potentiality to be a new food crop of the future owing to being a source of genes for resistance against biotic and abiotic stresses, the genetics of the domestication process in moth bean is not known. Its domestication will principally involve phenotypic changes, including reduction of seed dormancy and pod shattering, increased organ size and earlier flowering and maturity, which needs extensive knowledge of its genomic resources that are currently scarce. Recently, Yundaeng et al. (2019) constructed a genetic linkage map for moth bean, based on an F_2_ population of 188 individuals produced from a cross of wild moth bean (TN67) and cultivated moth bean (ICPMO056), and utilized it for the identification of quantitative trait loci (QTL) for domestication-related traits that can be used for genetic improvement of the moth bean and related *Vigna* species. The genetic linkage map comprised of 11 linkage groups (LGs) of 172 simple sequence repeat (SSR) markers that spanned a total length of 1016.8 centimorgan (cM), with an average marker distance of 7.34 cM. Additionally, a high genome synteny was observed between moth bean and other orphan legumes; mung bean (*Vigna radiata*), adzuki bean (*Vigna angularis*), rice bean (*Vigna umbellata*), and yard long bean (*Vigna unguiculata*) based on comparative genome analysis. A total of 50 QTLs and 3 genes associated with 20 domestication-related traits were identified. Most of the QTLs belonged to five LGs (1, 2, 4, 7, and 10). Pod shattering, trailing plant canopy, seed dormancy, poor yield potential, less responsive to input resources, late flowering and maturity are the key attributes of moth bean which indicates that the crop is still under active domestication. In another study, Somta et al. (2018) developed QTL mapping in F_2_ population (188 plants) of moth bean by crossing resistant accession, ‘TN67’ (male parent) and susceptible accession, ‘IPCMO056’ (female parent) for seed resistance to adzuki bean weevil (*Callosobruchus chinensis* L.). Segregation analysis suggested that *C. chinensis* resistance in TN76 is controlled by a single dominant gene, designated as *Rcc*. QTL analysis revealed one principal and one modifying QTL for the resistance, named *qVacBrc2.1* and *qVacBrc5.1* respectively. *qVacBrc2.1* was located on linkage group 2 between simple sequence repeat markers CEDG261 and DMB-SSR160 and accounted for 50.41 to 64.23% of resistance-related traits, depending on the trait and population, while *qVacBrc5.1* was detected in only one population. Somta et al. (2018), suggested that markers CEDG261 and DMB-SSR160 should be useful for marker-assisted selection for *C. chinensis* resistance in moth bean. A list of genetic linkage maps in *Vigna aconitifolia* is depicted in **Table 7**. Oliveira et al. (2020) presented the first physical map of moth bean and compared chromosomes with other *Vigna* and *Phaseolus* species. As with Yundaeng et al. (2019), a high magnitude of genome synteny was observed between moth bean and other related pulse crops such as mung bean (*Vigna radiata*), adzuki bean (*V. angularis*), rice bean (*V. umbellata*), yard long bean (*V. unguiculata*) as well as common bean (*Phaseolus vulgaris*) (Oliveira et al., 2020). As a result, molecular markers and genomic resources developed in these crops will be highly useful in moth bean crop improvement programmes.

**Table 7:** List of genetic linkage maps in *Vigna aconitifolia*.

Name of population	Trait	Gene/QTLs	PV explained by the QTLs (%)	Linkage group	References
IPCMO056 × TN67	*Callosobruchus chinensis* L. resistance	*Rcc*	-	-	Somta et al., 2018
		*qVacBrc2.1*	50.41 - 64.23	2	
		*qVacBrc5.1*	12.19	5	
TN67 × ICPMO056	Domestication related quantitative traits	*Pdt1.1-, Npdd1.1-, Sdwa1.1+*, *Sd100wt2.1+, Sdl2.1+*, etc.	3.72 - 68.67	1, 2, 3, 4, 6, 7, 8, 9, 10	Yundaeng et al., 2019

### 7.2 Transcriptome analysis

An extensive study was done to identify genes associated with moisture stress tolerance utilizing differential transcriptome assembly under stressed and non-stressed environments (Tiwari et al., 2018). They have identified 51 differentially expressing transcripts along with 1287 useful SSRs (Simple sequence repeats). Another study was done to identify genes associated with heat stress utilizing forward suppression subtractive hybridization (SSH) cDNA library of heat tolerant cultivar RMO-40 (Gurjar et al., 2014). A total 125 unigenes, out of which 21 were novel to moth bean, were identified. Functional annotation of ESTs (expressed sequence tags) led to the identification of enzymes and heat-shock proteins involved in plant defense against heat stress. In a first ever attempt to identify heat stress responsive genes in moth bean, an elevated temperature of 42°C for 5 min was given to a heat tolerant moth bean cultivar RMO-40 (Rampuria et al., 2012). Utilizing SSH cDNA library, 488 unigenes were identified that were constructed from 738 ESTs. Annotation and semi-quantitative PCR analysis indicated 20 signaling genes and 16 transcription factors associated with heat stress in moth bean. Recently, a number of transcriptome studies in other *Vigna* species have been added to the public data bases. Transcriptome derived resources, particularly EST-SSR markers, from other *Vigna* species can be utilized in genetic mapping, marker trait association and marker assisted selection for improvement programmes in moth bean.

### 7.3 Genetic transformation

Direct transformation was found to be an efficient transformation method in moth bean (Köhler et al., 1987a). Protoplast was used to transform moth bean by using PEG (polyethylene glycol) treatment and electroporation method. The plant genotype also has the role in the transformation success rate (Köhler et al., 1987b). In another transformation method, 60% plating efficiency was observed using co-cultivation of protoplast cells with *Agrobacterium tumefaciens* containing the Ti plasmid derivative pGV38501103 (Eapen et al., 1987). Particle bombardment method was also successfully used for direct gene transfer in moth bean using mature embryos (Bhargava and Smigocki, 1994) and hypocotyl explants (Kamble et al., 2003). A stable transformation was reported by co-cultivation of primary leaves and cotyledonary nodes, as well as vacuum infiltration into cotyledonary nodes of moth bean (cultivars Jadia and Jawala), with *Agrobacterium tumefaciens* strain EHA105 accommodating the binary vector p35SGUSINT (Kumar et al., 2008; Kumar et al., 2010). Unlike the earlier studies, genotypic effect was not observed in transformation frequency in this study. However, other studies show that the methodology and genotypes influence the transformation frequency (Kamble et al., 2003).

## 8 Future perspectives

**Figure 6:**
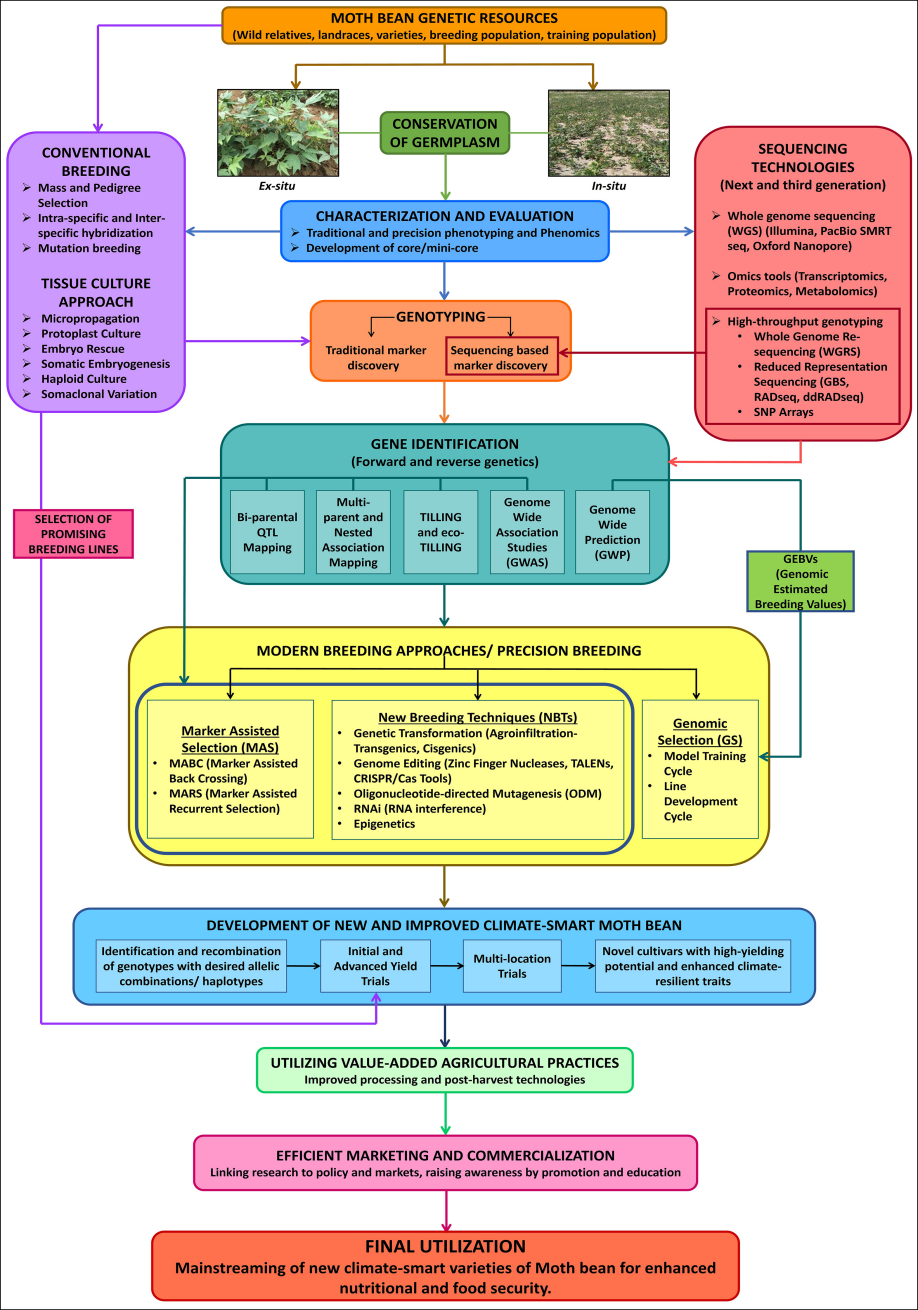
A blueprint for the mainstreaming of moth bean to develop climate-smart varieties.

The impact of drastic changes in global environment on crop productivity has been recognized by the agricultural sector, resulting in increased awareness about Climate Smart Agriculture (CSA) approach in order to increase food security and mitigate the effects of climate change. Orphan crops, though neglected, are well acclimatized to current conditions. Moth bean has evolved and is adapted to hot arid and semi-arid agro-climatic conditions, which makes it a relevant crop amid such challenging situations, with the potential to serve as an alternative to major legume crops. However, owing to its agronomic constraints, cultivation of the crop is still confined to traditional growing areas. Moth bean has not been fully exploited and has also lagged behind other pulses in terms of the quantum of developed genetic and genomic resources. Since there is limited number of genetic resources, considerable efforts are needed to carry out extensive explorations as well as characterization and evaluation of the collected moth bean germplasm for employing the genetic variability for high yield potential, yield stability, improved salinity tolerance, multi-disease and pest resistance as well as enhanced nutrition. With respect to genomic resources, extensive efforts are needed for development of reference gold standard genome since it is fundamental to understand the genome composition and gene repertory of the crop. Further, whole genome resequencing (WGRS), transcripts and genome-wide SSR and SNP markers are required for the identification and annotation of novel genetic polymorphism/candidate genes which are responsible for different agro-morphological, biotic and abiotic stresses as well as biofortification traits, along with being crucial for the construction of genetic linkage maps and physical maps in the future. Therefore, by utilizing recent advances in breeding methods such as haplotype-based breeding, precision-based phenotyping and high throughput genotyping, accompanied with bioinformatic resources, in association with available genetic diversity can help in accelerating domestication in moth bean and expediting its productivity. Identifying pan-genome sequence variation associated with advantageous traits and utilizing them for development of varieties will have a greater impact. Neo-domestication of stress adapted wild species using mutation breeding and TILLING and *de novo* domestication through new breeding techniques (NBTs) including genome editing tools like CRISPR/Cas could also help in development of novel plants with desired traits (Tomooka et al., 2014; Zsögön et al., 2018). Additionally, utilization of innovative value-added agricultural practices besides adopting effective marketing strategies can be instrumental in the promotion of moth bean as a potential major legume crop (**Figure 6**). Concentrated, continuous and coordinated efforts are required, from both the scientific community and policy-makers, for implementing these approaches which will not only aid in the development of high yielding and climate-resilient varieties, but also in the mainstreaming of moth bean for re-diversification of global food systems. Finally, when these challenges will be addressed, moth bean, being nutritionally sound and environmentally hardy, is going to be a hopeful climate-smart legume crop for sustainable food and nutritional security across the globe.

## Author contributions

Conceptualization: KC and RC. Writing – Original draft preparation: KC, G, BT, and RC. Writing – Review and editing: RC and JR. All authors contributed to the article and approved the submitted version.
